# ‘This disease is not meant for the hospital, it is *Asram*’: Implications of a traditionally-defined illness on healthcare seeking for children under-5 in rural Ashanti, Ghana

**DOI:** 10.1371/journal.pgph.0000978

**Published:** 2022-09-08

**Authors:** Princess Ruhama Acheampong, Aliyu Mohammed, Sampson Twumasi-Ankrah, Augustina Angelina Sylverken, Michael Owusu, Emmanuel Acquah-Gyan, Timothy Kwabena Adjei, Easmon Otupiri, Ellis Owusu-Dabo

**Affiliations:** 1 School of Public Health, College of Health Sciences, Kwame Nkrumah University of Science and Technology, Kumasi, Ghana; 2 Department of Statistics and Actuarial Science, College of Science, Kwame Nkrumah University of Science and Technology, Kumasi, Ghana; 3 Department of Theoretical and Applied Biology, College of Science, Kwame Nkrumah University of Science and Technology, Kumasi, Ghana; 4 Department of Medical Laboratory Technology, College of Science, Kwame Nkrumah University of Science and Technology, Kumasi, Ghana; International Institute for Population Sciences, INDIA

## Abstract

Every child has the right to survive, grow and develop. However, in spite of the considerable global gains that have been made in child survival, Sub-Saharan Africa still has the highest child mortality rates and accounts for the greatest burden of mortality globally. The majority of these children die without ever reaching a health facility. The practice of appropriate healthcare-seeking behaviour has a great potential to reduce the occurrence of severe and life‐threatening childhood illnesses. Several factors, however, influence healthcare-seeking behaviour, including perceptions of the cause of illness and socio-cultural perspectives. This study seeks to understand local concepts of a traditionally-defined illness complex, *Asram*, and its influence on healthcare seeking behavior of mothers/caregivers. This qualitative study was conducted from October 2019 to February 2020. Four Focus Group Discussions were conducted with mothers/caregivers of children under-5 and 22 Key Informant Interviews with mothers/caregivers of children who had *Asram*, health workers at district, facility, and community levels, and *Asram* healers. Participants were selected from two rural communities, Akutuase and Wioso of the Asante Akim North district in the Ashanti region of Ghana. Data analysis was carried out iteratively throughout data collection, using a thematic analysis approach. The study shows that *Asram* is a childhood illness complex that is perceived to have been acquired spiritually and/or inherited. Nine types of *Asram* were described. This childhood illness was said to be treatable by *Asram* healers who had sub-specialties in treatment approaches that were determined by the *Asram* type reported. Mothers/caregivers trusted *Asram* healers and preferred to call on them first. This was found to be the main reason for delays in seeking healthcare for children under-5 who showed symptoms of *Asram*. *Asram* is a childhood illness complex that is believed to be better managed outside the health facility setting. This study complements existing knowledge and creates opportunities for further research and the introduction of more effective interventions in the effort to improve child survival in rural communities.

## Background

One of the fundamental rights of every child is to survive and thrive [1], but sadly, 5.3 million children died in 2018 before reaching their fifth birthday [2] due to avertible causes [3]. Although considerable efforts in child survival have been made across the globe, Sub-Saharan Africa (SSA) still accounts for the highest child mortality rates [4]. A huge number of these children die without ever reaching a health facility [5].

The 2014 Ghana Health Demographic Survey showed that under-5 mortality rates were higher among children from rural areas when compared to urban areas [6]. Even with Ghana’s comprehensive health policies, concerted efforts, and community interventions to improve child health outcomes, countless children under-5 living in rural communities still die at home without seeking appropriate care [3].

The practice of appropriate healthcare seeking behaviour has a great potential to reduce the occurrence of severe and life‐threatening childhood illnesses. Recognizing the importance of healthcare seeking, the World Health Organization (WHO) and the United Nations Children’s Fund (UNICEF) highlighted activities to improve family and community health practices (including disease recognition and care seeking) as one of the three central components of the Integrated Management of Childhood Illness (IMCI) strategy [6]. However, many factors influence healthcare seeking behaviour, including perceptions of the cause of illness, socio-cultural perspectives, distance, cost, and quality of available care [7, 8].

Extensive literature has been published concerning healthcare seeking behavior of caregivers in SSA, however, only a handful of studies have considered the effects of local concepts of illnesses and traditionally-defined illnesses such as *Asram* (a traditionally-defined childhood illness complex in Ghana, which has been linked to spiritual causes, rather than physical or biomedical), on choices of healthcare. Those who have attempted to look at traditionally-defined illnesses have looked mainly at children suffering from malaria [9–11].

Few studies have made attempts to look at locally-defined illnesses in Ghana and their effect on neonatal mortality [7, 12], however, literature on the effect of traditionally-defined illnesses on general under-5 mortality is scanty and largely unavailable. This study, therefore, looks at the implications of a traditionally-defined illness, *Asram*, reported to be a major cause of neonatal and under-5 mortality in the Asante Akim North district of Ghana.

*Asram* has been defined elsewhere as a super-diverse illness, which is treated in communities, and perceived as ‘not for the hospital’. Some studies have also described Asram as a spiritual disease that can be passed on from a pregnant woman to an unborn child [12, 13]. Although *Asram* does not seem to have a biomedical equivalent, previous studies have described it as potentially severe [13].

Understanding local concepts of this traditional illness and its influence on healthcare seeking behaviour can complement existing knowledge and create opportunities for further research and the introduction of more effective interventions in the effort to improve child survival, growth, and development in rural Ghana.

## Methods

### Study design

This was a qualitative study that employed Focus Group Discussions (FGDs) with mothers/caregivers of children under-5 and Key Informant Interviews (KIIs) with health workers at both facility and community levels and *Asram* healers.

### Setting

We carried out the study in two rural communities of the Asante Akim North district of Ghana. The Asante Akim North District is one of the twenty-seven (27) districts of Ashanti, Ghana. It is located on Latitude 6 ° 37ꞌ 2.99ꞌꞌN and Longitude: -1 ^ᵒ^12ꞌ 21.60 ꞌꞌ W. The total population of the District is 69,186. The district has a more populous rural sector (53.5%) than the urban sector (46.5%). They are predominantly farmers and have eight health facilities, including 3 Community-Based Health Planning and Services (CHPS) compounds. Two rural communities were purposively selected for this study; Akutuase and Wioso.

Wioso has a population of 2,372 with 613 households and 369 children under-5. On the other hand, Akutuase has a total of 2,217 people with 502 households. Its children under-5 were enumerated to be 325. Both communities are found to the west of the district capital, Agogo.

The selection of these communities involved two steps; the first part of the process was on findings from a larger study that employed a positive deviance approach to gathering information from communities in the Asante Akim North district for a health education intervention. Four communities, Agogo, Pataban, Akutuase, and Wioso largely attributed child morbidity and mortality to traditional illnesses during the FGDs. However, based on the definition of rural communities in Ghana, as communities with less than a population of 5000 [14], Akutuase and Wioso were eligible.

Additionally, consultations were made with the District Health Directorate and Community Health Volunteers (CHV) who affirmed these two rural communities as having the most sought-after *Asram* healers in the district. To protect the privacy of study participants, Akutuase was referred to as community 1 and Wioso, community 2.

### Participant selection

The authors adhered to the qualitative reporting standards by following the 32-item consolidated criteria for reporting qualitative studies (COREQ) checklist [S1 Appendix].

As shown in Table 1, a total of 69 individuals, including 47 caregivers/mothers in 4 Focus Group Discussions and 22 in Key Informants Interviews (KII) were selected.

**Table 1:** Sample of KIIs and FGDs.

Sampling unit	Number sampled
**Key Informant Interview (KII) participants**	
Caregivers of children under-5 with *Asram*	4 (2 from each community)
Health workers at the facility level	1 District Nutrition Officer, 2 Nurses, 1 midwife
Health workers at the community level	2 CHPS Workers, 2 Community Health Volunteers (one in each community)
*Asram* healers	10 (5 in each study community)
**Focus Group Discussions (FGD)**	
Akutuase Community	2 (FGD 1: N = 12; FGD 2: N = 11)
Wioso Community	2 (FGD 1: N = 12; FGD 2: N = 12)

### Ethical considerations

The Committee for Human Rights and Publication Ethics (CHRPE) of the Kwame Nkrumah University of Science and Technology/Komfo Anokye Teaching Hospital approved the study (CHRPE/AP/637/19). Researchers explained the study objectives to prospective participants and assured them of confidentiality and anonymity. They sought their voluntary consent to partake in the study. After the informed consent process, participants were made to sign or thumbprint two consent forms; one for the researchers and one for the participants. Witnesses also signed the consent forms.

Verbal consent was also sought before recording each interview or discussion. Discussants and interviewees were informed that their voices would be recorded as they affirmed to be part of the interview or discussion.

The District Health Directorate provided administrative clearance for the study to be carried out in their district.

#### Focus Group Discussions (FGD)

Participants were selected using the purposive sampling method. The selection criteria for caregivers were; residents in the study communities; a mother/caregiver with a child under the age of 5; aged 18–49 years, consenting to participate in the study.

Twenty-three mothers/caregivers were eligible from Akutuase Community, whilst 24 were eligible in Wioso. Participants were put into one of two FGD groups in each community; 1 and 2 at random. No participant dropped out of the study in both communities throughout the study.

#### Key Informant Interviews (KII)

In recruiting mothers/caregivers and *Asram* healers for KIIs, snowball sampling was used. This involved recruiting key informants identified through other initial study participants [15] who were purposively selected. The purposive selection of the initial study participants followed an eligibility criterion for caregivers who had children with the *Asram* condition and *Asram* healers who were still in business and within the catchment area.

Health Workers within the facilities and communities were also purposively selected based on their constant interaction with mothers/caregivers who report the *Asram* condition. These participants were purposively sampled to ensure that respondents with an extensive understanding of the *Asram* disease participated in the study.

### Study population

As shown in Table 2 below, a total of 69 mothers/caregivers, *Asram* healers, health workers, and volunteers took part in the study. About 13% of study participants were aged 50 and above and the rest were 18–49 years. The majority had primary education (66.7%) and were farmers (47.9%). Forty-four participants were married and 39 were of the Akan tribe. The majority (49.3%) had a child under-5, with 76.8% being Christians.

**Table 2:** Socio-demographic characteristics of the study population.

Characteristics	N	Percent (%)
**Age of caregivers**		
18–49	60	87.0
50 and above	9	13.0
**Level of Education**		
Tertiary	3	4.3
Secondary	8	11.6
Primary	46	66.7
No formal education	12	17.4
**Occupation**		
Farmer	33	47.9
Trader/Apprentice	19	27.5
Salaried worker	7	10.1
Unemployed	10	14.5
**Marital Status**		
Married	44	63.8
Single	25	36.2
**Ethnicity**		
Akan	39	56.5
Ga/Dangbe	4	5.8
Ewe	2	2.9
Mole-Dagbani	6	8.7
Grusi	8	11.6
Gurma	8	11.6
Other	2	2.9
**Number of children under-5**		
None	10	14.5
1	34	49.3
2	18	26.1
3	7	10.1
**Religion**		
Christianity	53	76.8
Islam	12	17.4
Other	4	5.8

Overall total = 69 participants Total Number of mothers/caregivers in FGD and KII = 51

### Data collection

This study was conducted from October 2019 through February 2020. Three main categories of participants were interviewed: mothers/caregivers, *Asram* healers and health workers at community and facility levels.

A saturation point, defined as the phenomenon in which additional sampling units are not yielding new information [15], was used to determine the ultimate sample size. Research Assistants debriefed each other after each FGD and KII and decided when data saturation occurred. Consequently, 4 FGDs and 22 KIIs were conducted face-to-face.

FGDs were held with women of reproductive age (WRA), aged 18–49 years who had children less than the age of five. Three FGD groups had 12 participants whilst one group had 11 participants. In all, 47 mothers/caregivers were sampled for the FGDs.

KIIs were conducted among *Asram* healers, caregivers who had ever had or presently had children with the ‘*Asram*’ condition, Community Health Volunteers (CHVs), nurses, a District Nutrition Officer, and a midwife.

An FGD guide [S2 Appendix] and 3 KII guides: mothers/caregivers [S3 Appendix], *Asram* healers [S4 Appendix], and health workers [S5 Appendix] were developed by authors and pretested in two rural communities with characteristics similar to the study communities; Dwendwenase and Boankra. The guides were written in English and translated into Twi and back to English by experts. The final interview guides were accepted by the team before four Research Assistants (RAs) were trained in qualitative research methods to conduct the interviews. The RAs were made up of a Ph.D. student and three master’s students. They were made up of two males and two females.

This combination of male and female RAs was because, in some rural areas which are gender-sensitive, a male participant might not be comfortable being interviewed by a female and vice versa. Discussions and interviews were held in places considered to be private for participants.

Discussions were done in the Twi language, as that is the main language of the study area, and discussants consented to use it.

Key Informant Interviews lasted for 30–45 minutes, while FGDs lasted for an average of 75 minutes. The Research Assistants did not have any prior relationship with study participants, and apart from the RAs and participants, no one was permitted to be a part of the discussions and interviews. This was done to encourage participants to share their perspectives on the *Asram* disease and to encourage group interaction during discussions. Notes were also taken during discussions and interviews to add additional context to the study.

### Data analysis

Audio recorders were used to record interviews and discussions. Recorded audios were immediately transferred to the computer and saved on a server, which was protected by a password. This was followed by verbatim transcriptions and data analyses.

Data analysis was carried out iteratively throughout data collection, using a thematic analysis approach [16]. This involved five main stages: 1) Familiarization with notes taken during interviews and discussions; listening to the recorded interviews and reading transcriptions. 2) Highlighting interesting phrases and sentences which were found relevant. Codes were then given to these phrases and sentences by authors PRA and EAG independently. Line by line, the data were categorized into analytic units under descriptive words. 3) Themes were then generated based on patterns that were identified with the codes generated earlier. 4) Themes were reviewed by comparing them to the main transcripts to make sure we were not missing anything. 5) The themes were then labelled and used in the write-up of the results.

Four major themes were identified: knowledge and perception of the *Asram* disease; causes and types of *Asram*; management of *Asram* and the implications of *Asram* on healthcare seeking behaviour for children under-5.

## Results

This qualitative study showed how common *Asram* is in the Asante Akim North district, and how much mothers/caregivers’ perception of this disease complex affects their healthcare seeking behaviour for children under-5 who show signs and symptoms of *Asram*.

### The context: Knowledge and perception of the *Asram* disease

Interviews and FGDs reported that *Asram* is perceived as a spiritual disease, which is ‘not meant for the hospital.’ *Asram* was described to be potentially dangerous and is a major cause of child under-5 mortality in the district.

*‘Asram is a spiritual disease that kills many children in this area*. *If a child dies in this community*, *you mostly find out that the child died of Asram*.’

*(65*-*year-old caregiver*, *KII)*

During all the FGDs, a major point raised by mothers/caregivers regarding their perception was that *Asram* is often passed on to children who are born very beautiful or handsome, hence, jealous people pass the disease on to the child.

*‘When I gave birth*, *my daughter looked very beautiful and plump*, *everyone seemed to like her*. *Suddenly*, *my daughter refused to suckle from my breasts*. *She cried incessantly at night and started reducing in weight*. *My mother drew my attention to the fact that a jealous neighbour had passed Asram on to my baby*. *I did not doubt that woman had done that*! *Sadly*, *my baby passed on when she was 6 months old*.*’*

*(35-year-old mother*, *FGD)*.

More interestingly, mothers/caregivers who had utilized a Child Welfare Clinic (CWC) services and were participants of the FGDs revealed that one of the major reasons why mothers/caregivers refused to bring their children to the CWC for growth monitoring was because children who weighed better and were commended by health care workers became a target for evil persons who were also around to pass the disease onto innocent children. Subsequently, those children have a sharp reduction in weight the following month, even if they did not appear ill.

*As for me*, *I bring my child for weighing after the last person has left and the nurses are sweeping*. *I do this because this is one of the points where people pass Asram onto our babies*. *I learnt my lessons after my first child nearly died of Asram*.

(*32-year-old mother*, *FGD*)

It was further revealed that *Asram* healers are perceived to have the power to heal and also cause a child to have *Asram*, hence, they are not allowed to see babies.

‘*Even when my daughter delivered*, *she hid my grandson from me*. *I did not attend the naming ceremony of the baby too*. *Immediately I see the baby*, *he might start showing some signs of the Asram disease*.’

*(75-year-old Asram healer*, *KII)’*

Another perception noted in this study was the fact that *Asram* was hereditary and could be passed on from a parent to a child. Some caregivers said *Asram* was found in particular families and could occur as new children are born into that family.

### Causes and types of *Asram*

Results of interviews showed that *Asram* was categorized depending on the signs the sick child presented with. The types of *Asram* were described during interviews, the next sections, therefore, describe the types of *Asram* as were gathered through FGDs and KIIs:

#### Asram boredwo

*Asram boredwo* (*boredwo* is translated as roasted plantain) is the type of *Asram* in which the child is emaciated and has a relatively bigger head than the body with dried-up skin. This *Asram* is said to be acquired during pregnancy. It was revealed that if a pregnant woman eats either roasted plantain or fried plantain during pregnancy, she often contracts this form of *Asram* in pregnancy, thereby giving birth earlier than expected, and consequently having a baby who is unable to grow well, looking as dried up as roasted plantain.

It was reported that a pregnant woman could potentially expose her child to *Asram* if she dressed up attractively with jewelry and exposed certain parts of her body such as her thighs, stomach, arms, and backside. Dressing in a manner perceived to be indecent while pregnant is severely abhorred in the area, so respondents also saw this as a punishment for women who did not comply with such cultural rules. *Asram boredwo* was concluded to be a punishment for a stubborn pregnant woman.

*‘Some pregnant women are so stubborn*! *They go about dressing in a manner that draws public attention*. *If you observe*, *you will see that they are those who often bring their children to me for treatment after delivery*.

*(72-year old Asram healer*, *IDI)*

*Asram boredwo* is also believed to be caused spiritually by *Asram* healers. They are not allowed to come into contact with babies because babies easily have the symptoms of *Asram boredwo* as soon as they are seen by a healer. Babies are therefore hidden from these healers.

It was also revealed by an *Asram* healer who had been treating children for over 30 years that *Asram boredwo* was the most common type and easily killed children before their first birthday.

#### Asram ntoos

*‘Ntoos’* translates in English as ‘tomatoes’. As the name implies, *Asram ntoo*s is described as multiple blisters on a baby’s skin, looking like roasted tomatoes.

This type of *Asram* is believed to be the result of a pregnant mother walking through a spider web or eating squirrel meat during early pregnancy. Others believed if a woman ate excessively hot meals during lactation, it made breast milk overly hot for babies, and thereby giving them the ‘tomato-like’ blisters.

‘*As for Asram ntoos*, *we women allow them on our children*. *You can see a pregnant woman craving the meat of a squirrel*. *We don’t have many squirrels around*, *but they will find means and ways of getting them from other communities*. *What sort of craving is that*?

*(27- year old caregiver*, *FGD)*

#### Asram borfre

*Borfre* is translated into English as *pawpaw*. When a child’s head is swollen and bigger than normal, it is *Asram borfre*. The child is also observed to have softer skin than normal. This type is believed to be a punishment for a pregnant woman who ate pawpaw during pregnancy. This type was said to be common among children who were born before the ninth month. Children who had *Asram borfre* were also described as children who cry a lot and barely make their mothers sleep.

#### Asram mpompo

*Mpompo* is translated as boils in English. When children have rashes all over their bodies, it is referred to as *Asram mpompo*. *Asram mpompo* was revealed to start with a few rashes on the skin or face but gradually spreads to all parts of the body. The rashes, as described, are reddish at times and could cause fever in children. *Asram mpompo* was said to be one that would hardly cause the death of a child.

#### Asram mpaemu

*Mpaemu* refers to the fissures in the skull. It was revealed that a child who had *Asram mpaemu* had the skull split into two to four parts. A*sram mpaemu* was believed to cause a downward and upward movement near the fontanel of a baby. This, they described as a severe form of *Asram mpaemu*.

#### Asram ayamtuo

*Ayamtuo* is translated in English as diarrhoea. Some KIIs revealed that with *Asram ayamtuo*, children had bloated stomachs within the first weeks after birth and usually have watery stools. It was revealed that they often have green veins on their bloated stomachs, causing them to cry incessantly, especially at night. Such children were described as ‘cry babies’ which resulted in them losing weight, especially in their first six months.

#### Asram esuro

*Esuro* means convulsions in English. *Asram esuro* is when a child, normally between the ages of one and five suddenly has a spike in temperature and convulses with clenched fingers and with the eyes widely open. When a child has *Asram esuro*, he/she is not supposed to be carried by the mother or any other woman. It is believed that the child is most likely to die if handled by a woman.

*Asram esuro* is described as one of the easiest to treat by *Asram* healers, hence, one of the cases reported to them earliest.

#### Asram nofo-denden

*Nofo-denden* is translated as engorged breasts in English. *Asram nofo-denden* was described as an illness that made a mother’s breasts ‘hard’ and ‘hot’ with hardened nipples. This makes a baby unable to suckle, especially during the first few days after delivery. Caregivers, during FGDs, described this *Asram* as one that is painful and is believed to be acquired during pregnancy and passed on to a baby.

It was also revealed that caregivers who cheated on their partners during pregnancy suffered from this type of *Asram*, compelling their children to lose weight in the first weeks and some eventually dying after unsuccessful treatments from the *Asram* healer.

#### Asram pepe

*Pepe* refers to fast breathing in English. Responses from interviewees showed that *Asram pepe* was one of the most dangerous types of *Asram* since it often results in neonatal mortality.

‘*The symptoms of Asram pepe are terrifying as you can see the baby either struggling to breathe or breathing excessively fast*. *You can just see the Asram holding the ribs*, *making the child suffer to breathe*. *Before you know*, *your baby is dead*! *It happened to me*’.

*(33-year-old mother*, *KII)*

A mother who had lost her baby to *Asram pepe* attributed hers to prolonged delivery. She explained that she was in labour for over 48 hours, having contractions stopping intermittently until she finally delivered a baby who was too exhausted to cry. She described her baby as having a colour like a sky (blue). The baby, she mentioned died about two hours after delivery.

#### Management of *Asram*

*Asram* is mainly managed by special healers in each community. The members of the community are aware of the various symptoms that require the attention of an *Asram* healer. Right from the day of delivery, mothers run to *Asram* healers when they notice any changes such as the inability of a baby to breastfeed, fast breathing, yellow eyes, sunken fontanel, greenish stools, rashes, boils, watery stools, and fever.

‘*In this community*, *there are certain symptoms we look out for when we give birth or when our children are growing*. *Once you see a child unable to breastfeed*, *crying incessantly*, *passing yellowish or greenish stools*, *then you just know it is a disease that is not meant for the hospital*, *because*, *it is Asram*!’

(Caregiver, 44-year-old caregiver, FGD)

*Asram* healers use traditional herbs which are not disclosed to anyone, not even a spouse. It was revealed through interviews with some *Asram* healers that the combination of herbs and materials used for the treatment is passed on from generation to generation.

### Baptism with a chameleon

A child under-5 is taken through a process called baptism before the treatment process starts. Healers disclosed that a chameleon is killed and mixed with three ‘undisclosed’ herbs. Findings from this study showed that chameleon baptism is believed to be the most powerful treatment for *Asram*.

A caregiver who was also identified to be an *Asram* healer’s wife revealed the process of baptism as;

‘*He collects materials from three different specific trees from the bush*, *mixes and preserves them in a bucket*. *He then buys* “*adaadanho*” *(chameleon) meat (keeps it under the bucket)*. *He washes the trees in a big pan and keeps them in a bucket again with water and preserves it for seven days*. *You then bathe the baby with it for at least three days and he’ll get better*.

*(62 year- old Asram healer’s wife*, *KII)*

### The secret ‘wall gecko’ treatment

In a knowledgeable informant interview with a 75-year- old *Asram* healer, he reported that he uses a wall gecko to treat all types of *Asram*. It was further revealed that the wall gecko was killed, dissected, and hanged on a locally-made rope to dry for some days. The healer further explained that he would then boil the anal part of the dried-up wall gecko, and subsequently give a portion of the resultant concoction to a patient (mostly a child under-5) to drink.

It was also revealed that a portion of the dried-up wall gecko was hung around the neck of newborns as part of a locally-made necklace to ward off evil spirits or eyes which could potentially transfer *Asram* illness.

### The *Asram* bath therapy

Healers revealed that the most common form of treatment for *Asram* was giving a patient a hot bath with carefully selected boiled herbs and tree parts. Two out of the ten healers who were interviewed however mentioned that they specifically used cold water for their bath therapies because they worked better.

The herbal bath mixtures were revealed to be made up of either leaves, barks, roots, or branches of some trees. Others revealed that certain weeds were added to their bath therapies. Each type of *Asram* had specific combinations to suit the treatment for a specific number of days.

### *Asram* herbal mixtures

Healers treated *Asram ayamtuo* by using a mixture of two herbs grown only in the backyards of particular healers. This mixture of knowledge is passed on to the next healer when the current one is about to die.

Babies in their first few days are given such mixtures if they pass loose, greenish stools. Other healers mix the herbs with ginger as an enema used to cleanse the bowels of *Asram* children. This, they revealed causes the baby to immediately empty its bowels of any *Asram* trapped in its body.

“*When a baby starts having greenish stools during the first days*, *the mothers are often scared and run to me with the baby*. *I simply mix the herbs and give them to the child*. *Immediately the baby drinks this mixture*, *she passes extremely green loose stools*. *This shows that the intestines have been cleansed*.

*(58-year-old Asram healer*, *KII)*

It was also revealed that the bark of trees was given to mothers to boil to use as a medication for *Asram* children. However, this came with strict instructions; the firewood or charcoal to be used for preparing the medication should not be touched by anyone except the healer, else the *Asram* would be transmitted to the person’s household.

### Treatment period

A child who is diagnosed with *Asram* is often sent to an *Asram* healer in the community. The severity determines the number of treatment days. If the *Asram* is severe, the child stays in the house of the healer for some time, but if it is not severe, the mother/caregiver will take the child home and report each morning for treatment till the child recovers. As reported, *Asram esuro* (convulsion) is normally treated for four to seven days. *Asram boredwo* also takes a week, while *Asram ntoos* takes seven to nine days of treatment either in the home of *the Asram* healer or in the home of the mother/caregiver with strict guidelines.

‘*The doctors and nurses do not have a cure for Asram*. *I am surprised at those who take children who are convulsing to the hospital*. *Such children die in the health facilities because convulsion is the same as Asram esuro*, *and not for the hospital*!’

*(57-year-old Asram healer*, *KII)*

Healers proposed that after treatment, if a child is not getting better, then they recommended that the mother proceeds to the hospital.

‘*We ask the mother to take the child to the hospital if we do not see any improvement after the seven days of treatment*’

*(43-year-old Asram healer*, *KII)*.

A 60-year-old *Asram* healer also proposed that they be recognized by the Ministry of Health as a group for treating certain childhood illnesses.

‘*I would like to plead with you to inform the leaders of health that they should leave some convulsions for us to treat*. *It is Asram esuro*. *They have to leave it for us*. *When children from this community are admitted in the hospital for convulsions*, *they do not come back*’

*(60-year-old Asram healer*, *KII)*

### Payment

It was disclosed that mothers/caregivers are not obliged to pay any money after their child is treated. Mothers/caregivers had a choice to give an offering or a gift such as a goat, sheep or fowl which they termed ‘*aseda’ (thanksgiving)*. This was found to be an incentive when compared to the amount they had to pay to reach the health facility or pay within the facility.

‘*Payment terms are so flexible and cheaper when we run to Asram healers*. *I struggle to pay my bills at the hospital because I do not have insurance*. *So why do I have to worry when the Asram healer is here*?

*(34-year-old caregiver*, *FGD)*

### Implications of *Asram* on healthcare seeking behaviour of caregivers with children under-5

*Asram* is regarded as an illness that requires the attention of the famous *Asram* healers for a number of days and children are only referred to the hospital based on their orders.

Healthcare workers, through KII, described *Asram* as the same danger signs of childhood illnesses they educate mothers on. They further revealed that mothers, especially those with neonates, believed *Asram* healers could heal their babies better than the healthcare workers did, so they would often come to the health centres when they find out that the child is almost dying.

‘*It is really unfortunate*. *We suffer as health workers in rural areas because they do not listen to us*. *Every sickness is Asram*! *We are tired*!. *When a child is convulsing*, *Asram; when a child has diarrhoea*, *they say it’s Asram; fever*, *Asram… Just imagine*, *when a baby is unable to feed*, *they say he/she has Asram*! *Losing weight*, *Asram*…*aaba*! *Every sickness is Asram*! *It’s so discouraging*!

*(33-year-old nurse*, *KII)*

A nutrition officer, through the interview, mentioned that children die of malnutrition in the district because when a child is unable to breastfeed or eat well, mothers/caregivers consider it as *Asram boredwo* and proceed to the *Asram* healer. The healer then gives the baby some herbs which mostly leads to diarrhoea and further loss in weight. They are said to mostly report when the child is found weak and sometimes unresponsive. He added that *Asram nofo-denden* was breast engorgement, and instead of seeking appropriate care so that the baby could suckle, mothers/caregivers resort to *Asram* healers, causing the babies to lose weight and eventually have to be admitted to the nutrition rehabilitation centre for Kwashiorkor. It was revealed that children often recover and gain weight when they are given plumpy nuts, nutritional supplements and when breastfed often.

*‘A boy of almost two years weighed 6*.*5 kg when he was admitted*. *They claimed it was Asram*, *but after a few weeks of good feeding and constant monitoring*, *the boy left the rehab weighing 11kg*. *It’s sad a lot of these innocent children die of hunger and malnutrition*, *yet they keep them at home*, *claiming it is Asram*.

*(27-year-old nutrition officer*, *KII)*

Interestingly, a midwife mentioned during her interview, that at some point, she almost believed in the *Asram* treatment because this illness was constantly mentioned whenever a baby was delivered. She further explained that preterm babies and underweight children were immediately called *Asram* patients right from the delivery ward by their mothers.

‘*I was shocked to hear mothers themselves cry*, *not because of the weight or condition of their babies*, *but because they would be called Asram patients and would have to spend some days with an Asram healer*’

*(31-year-old midwife*, *KII)*

Community Health Volunteers (CHV) who were interviewed had a strong belief in the *Asram* disease complex and did not see better treatment from the hospital as compared to the *Asram* healers in their communities.

‘*Asram is real*. *There is no hospital treatment for Asram*, *and those sent to the hospital mostly die there*’

*(56-year-old CHV*, *KII)*

It is interesting to find out that during FGDs, caregivers/mothers mentioned that nurses had advised them to leave the health facility and go home for traditional medicine, this was confirmed by a Community Health Volunteer (CHV), who had this to say:

‘*Madam*, *there is Asram*! *I believe it*! *As for Asram*, *I also hold the view that it is not meant to be treated at the hospital*, *because*, *they cannot treat it*, *even some of the nurses understand this*’

*(62-year old CHV*, *KII)*

Another CHV also affirmed that only the *Asram* healers could heal *Asram*.

‘*Asram esuro (the one that makes children convulse) for example*, *I confidently tell the caregivers to go to an Asram healer in my community*. *He treats it best*’

*(55-year-old CHV*, *KII)*

A caregiver admitted that she believed in the fact that *Asram* was a major cause of morbidity and mortality among children under-5 in her community, but she mentioned it had dire consequences.

*I believe there is Asram*, *but I believe if we go to the hospital first*, *they can treat Asram*. *The other time*, *I refused to go to the Asram healer*, *against my husband’s advice*, *and my child recovered quicker than usual*. *In a way*, *I am beginning to think that Asram can be treated better in the hospital*’

*(28-year-old caregiver*, *KII)*

## Discussion

Prompt and appropriate healthcare seeking practices for children under-5 are essential in the reduction of deaths resulting from delays and seeking inappropriate healthcare in developing countries [17]. As in previous studies in Ghana [7, 12], this study found that healthcare seeking behavior of mothers/caregivers is a complex process, which is strongly affected by health beliefs. Besides, local illness concepts have been described to be related to inappropriate healthcare-seeking behavior in SSA, and often led to child mortality [18].

The findings of the current study expose the potential barriers to healthcare seeking for the traditionally-defined childhood illness *Asram*, and its implications on the reduction in infant and child mortality in rural Ghana. This study equally proves that *Asram* is a likely barrier, not only to prompt health care seeking for children under-5 in Ghana but also to the achievement of the third Sustainable Development Goal (SDG), which seeks an end to preventable deaths of newborns and children under-5 [4].

This qualitative study confirms that *Asram* is a common traditionally-defined illness labelled as ‘not meant for the hospital’ as previous studies in Ghana have described. *Asram* is classified based on the description of its signs, symptoms, and causes. The nine different types of *Asram*: *boredwo*, *ntoos*, *mpaemu*, *ayamtuo*, *mpompo*, *nofo-denden*, *mpaemu*, *pepe*, *and esuro* were described as causes of child morbidity and mortality in the study communities. A similar study [12] carried out in the Kintampo North and South districts, however revealed 17 different types of *Asram* from multi-ethnic groups, including northern migrants in the two districts.

*Asram* is believed to have different meanings and names in different parts of Ghana among the different ethnic groups [19, 20]. This could have been a reason for the difference in types of *Asram* described in the Bono and Ashanti regions of Ghana. For example, *Asram ntoos*, *Asram mpompo*, *Asram boredwo*, *Asram pepe*, and *Asram esuro* had similar characteristic symptoms as found in our study. Others like *Asram nofo-denden* and *Asram kwashiorkor* had similar characteristics but different names in the study conducted in Kintampo. Interviews with more than two communities could have revealed other types which were hitherto not found by this study.

This study further showed that *Asram* is treated by special healers who inherit the position from a close relative. *Asram* healers are the first point of call when a child is sick and is labeled as an *Asram* patient. Children under-5 diagnosed with *Asram*, often die in the communities and these deaths are not reported to the health facilities. Others are sent to the hospital after days of delay and unsuccessful treatment by *Asram* healers. This finding is no different from Hill et al, who asserted that children diagnosed with local illnesses such as *Asram* are severely ill and that traditional beliefs are a significant barrier to appropriate care seeking in rural Ghana [7]. Other studies in parts of Africa, [18, 19, 21, 22] have reiterated the potential threat of traditional illnesses, which are labeled as not-for-hospital, are to efforts to reduce infant and child mortality through prompt and appropriate healthcare seeking [23].

*Asram*, as described, has dire implications on child survival in rural areas and strategies must be designed to overcome barriers to healthcare seeking for children in these vulnerable communities. It is also important to acknowledge and appreciate the role of *Asram* and other traditionally-defined illnesses in unaccountable deaths which occur in rural communities [24]. Our study showed a strong belief of mothers, and CHVs in *Asram* healers, and their preference for their treatments of herbs and bath therapies; other studies report similar findings [18, 25].

The strength of this study borders on the rigour involved in data collection and analysis and the depth of knowledge gathered from knowledgeable informants. The lack of generalizability, however, is a limitation, although it is quite common with qualitative studies. Though two rural communities were used in this study, the findings can be applied to other rural settings in the Ashanti Region of Ghana. That notwithstanding, a larger sample size drawn from both rural and urban communities could have provided additional critical information on the role of traditionally-defined illnesses on child survival in Ghana.

## Conclusion

Despite the complexity in the meaning and perceptions related to *Asram* locally, it is possible to identify the categories of illnesses medically. Relating this to timely healthcare seeking, healthcare workers should capture *Asram* and other traditionally-defined illnesses into the design of social and behaviour change communication interventions that target pregnant women and caregivers of children under-5.

The study findings highlight the effects of traditionally-defined illnesses on prompt and appropriate health care seeking for children under-5 who are ill. Furthermore, it illustrates the importance of considering healthcare seeking behavior of caregivers from a wider context that considers the management of traditionally-defined illnesses such as *Asram* at the community level. Paying attention to, and understanding the context of traditionally-defined childhood illnesses and their implications on the achievement of the SDG 3 has become crucial. Future health interventions on health care seeking and survival of children under-5 should consequently consider education on traditionally-defined illnesses in rural communities and the dangers they pose.

Finally, this study suggests that *Asram* healers hold the belief that they are more entitled to treating these traditionally-defined symptoms, and policymakers must note this. It is, therefore, speculated that caregivers will continue seeking healthcare from these healers regardless of alternative biomedical advice. As such, the Ministry of Health should incorporate guidelines to appropriate healthseeking for children under-5 in the Ghana Child Health Policy. This, to an extent, could minimise the delays in seeking appropriate healthcare and in turn reduce under-5 mortality in Ghana.

## Supporting information

S1 Appendix Consolidated criteria for reporting qualitative studies (COREQ): 32-item checklist. (DOCX)

S2 Appendix Focus Group Discussion (FGD) guide for mothers/caregivers of children under-5 on the traditionally-defined illness, *Asram*. (DOCX)

S3 Appendix Key Informant Interview (KII) guide for mothers/caregivers of children under-5 on the traditionally-defined illness, *Asram*. (DOCX)

S4 Appendix Key Informant Interview (KII) guide for *Asram* healers on the traditionally-defined illness, *Asram*. (DOCX)

S5 Appendix Key Informant Interview (KII) guide for health workers/ community health volunteers on the traditionally-defined illness, *Asram*. (DOCX)

S6 Appendix Table summarizing the types of *Asram*, symptoms description, and treatment approaches. (DOCX)
