# Supplementary material for: ‘This disease is not meant for the hospital, it is Asram’: Implications of a traditionally-defined illness on healthcare seeking for children under-5 in rural Ashanti, Ghana
Source: PLOS Glob Public Health. 2022 Sep 8;2(9):e0000978. doi: 10.1371/journal.pgph.0000978 (PMC10021330; doi:10.1371/journal.pgph.0000978)
Supplement: S1 Appendix — (DOCX) [file pgph.0000978.s001.docx]

Consolidated criteria for reporting qualitative studies: 32-ITEM CHECHLIST

| **ITEM** |  | **Guide questions/description** | **Response / page number referenced** |
| --- | --- | --- | --- |
| Domain 1: Research team and flexibility  Personal characteristics | | | |
| 1. | Interviewer/facilitator | Which author/s conducted the interview or focus group? | Princess Ruhama Acheampong (PRA), Timothy Kwabena Adjei (TKA) and Emmanuel Acquah- Gyan (EAG) |
| 2. | Credentials | What were the researcher’s credentials? E.g. PhD, MD | PRA: MPH, BSc.  TKA: MPH, MBChB, MCPS  EAG: MPH, BSc. |
| 3. | Occupation | What was their occupation at the time of the study? | PRA: PhD student  TKA: MD and Research Fellow  EAG: Research Fellow |
| 4. | Gender | Was the researcher male or female? | *Data Collection: Paragraph 4 Page 12*  Males and females |
| 5. | Experience and training | What experience or training did the researcher have? | *Data Collection: Paragraph 4 Page 12*  All researchers were trained on qualitative research methods and were involved in a pilot study. |
| **Relationships with participants** | | | |
| 6. | Relationship established | Was a relationship established prior to study commencement? | *Data Collection: Paragraph 6, Page 12*  No relationship was established prior to the study. |
| 7. | Participant knowledge of the interviewer | What did the participants know about the researcher? e.g. personal goals, reasons for doing the research. | *Data Collection: Paragraph 6, Page 12*  Participants did not know the researchers prior to the interview |
| 8. | Interviewer characteristics | What characteristics were reported about the interviewer/facilitator? e.g. Bias, assumptions, reasons and interests in the research topic. | *Data Collection: Paragraph 6, Page 12*  No characteristics were reported. |
| Domain 2: Study Design  Theoretical Framework | | | |
| 9. | Methodological orientation and Theory | What methodological orientation was stated to underpin the study? e.g. grounded theory, discourse analysis, ethnography, phenomenology, content analysis | *Data Analysis:*  *Paragraph 1, Page 12*  Thematic analysis. Themes were developed from data collected during interviews |
| **Participant selection** | | | |
| 10. | Sampling | How were participants selected? e.g. purposive, convenience, consecutive, snowball | *Participant Selection:*  *(FGD) Paragraph 1*  *(KII) Paragraph 1 Pages 7, 8*  Purposive sampling for FGDs and  Purposive and snowball sampling for KIIs |
| 11. | Method of approach | How were participants approached? e.g. face-to-face, telephone, mail, email | *Data Collection: Paragraph 2, Page 11*  Face-to-face |
| 12. | Sample Size | How many participants were in the study? | *Data Collection: Paragraph 1, Page 11*  69 participants |
| 13. | Non-participation | How many people refused to participate or dropped out? Reasons? | *Participant Selection:*  *(FGD) Paragraph 1*  *Page 8*  None |
| **Setting** | | | |
| 14. | Setting of data collection | Where was the data collected? e.g. home, clinic, workplace | *Setting:*  *Paragraph 3 Page 7*  Private places chosen by participants. |
| 15. | Presence of non-participants | Was anyone else present besides the participants and researchers? | *Data Collection: Paragraph 6, Page 12*  No other person was present apart from researchers and participants |
| 16. | Description of sample | What are the important characteristics of the sample? e.g. demographic data, date | *Study Participants*  *Table 2* |
| Data Collection | | | |
| 17. | Interview guide | Were questions, prompts,  guides provided by the  authors? Was it pilot  tested? | *Data Collection: Paragraph 6, Page 11*  *(Additional files 2,3,4 and 5)*  Discussion and Interview guides were developed by authors and pretested for corrections before use in the main study. |
| 18. | Repeat interviews | Were repeat interviews carried out? If yes, how many? | Interviews were not repeated |
| 19. | Audio/visual recording | Did the research use audio or visual recording to collect the data? | *Data Analysis:*  *Paragraph 1, Page 12:*  Audio recorders and verbatim transcriptions |
| 20. | Field notes | Were field notes made during and/or after the interview or focus group? | *Data Collection: Paragraph 6, Page 11*  Field notes were made during the discussions and interviews and added additional context to the analytical process of the study. |
| 21. | Duration | What was the duration of the interviews or focus group? | *Data Collection: Paragraph 6, Page 12*  Key Informant Interviews lasted for 30 - 45 minutes, while FGDs lasted for an average of 75 minutes |
| 22. | Data saturation | Was data saturation discussed? | *Data Collection: Paragraph 2, Page 11*  Researchers debriefed each other after discussions and interviews and decided when data saturation occurred. |
| 23. | Transcripts returned | Were transcripts returned to participants for comment and/or correction? | Transcripts were not returned to participants |
| Domain 3: analysis and findings | | | |
| Data Analysis | | | |
| 24. | Number of data coders | How many data coders coded the data? | *Data Analysis:*  *Paragraph 2: Page 12*  2 authors, PRA, EAG coded the data |
| 25. | Description of the coding tree | Did authors provide a description of the coding tree? | No |
| 26. | Derivation of themes | Were themes identified in advance or derived from the data? | *Data Analysis:*  *Paragraph 2: Page 12*  Themes were identified from the data. |
| 27. | Software | What software, if applicable, was used to manage the data? | Microsoft excel and Nvivo |
| 28. | Participant checking | Did participants provide feedback on the findings? | No |
| Reporting | | | |
| 29. | Quotations presented | Were participant quotations presented to illustrate the themes / findings? Was each quotation identified? e.g. participant number | *Results:*  *Pages 13-24*  Quotations were presented to illustrate themes. Each quote was identified by participant’s age and means of data collection (FGD or KII) |
| 30. | Data and findings consistent | Was there consistency between the data presented and the findings? | *Results:*  *Pages 13-24*  Quotations were presented with interpretations for transparency between data and findings |
| 31. | Clarity of major themes | Were major themes clearly presented in the findings? | *Results:*  *Pages 12-24*  Four major themes were clearly presented in the findings Section. |
| 32. | Clarity of minor themes | Is there a description of diverse cases or discussion of minor themes? | *Page 25*  Discussion on varying results from other parts of Ghana were made in the discussion section. |

**Reference**

Tong A, Sainsbury P, Craig J. Consolidated criteria for reporting qualitative research (COREQ): a 32-item checklist for interviews and focus groups. *International Journal for Quality in Health Care*. 2007. Volume 19, Number 6: pp. 349 – 357
