# Supplementary material for: ‘This disease is not meant for the hospital, it is Asram’: Implications of a traditionally-defined illness on healthcare seeking for children under-5 in rural Ashanti, Ghana
Source: PLOS Glob Public Health. 2022 Sep 8;2(9):e0000978. doi: 10.1371/journal.pgph.0000978 (PMC10021330; doi:10.1371/journal.pgph.0000978)
Supplement: S2 Appendix — (DOCX) [file pgph.0000978.s002.docx]

**Focus Group Discussion (FGD) guide for mothers/caregivers of children under-5 on the traditionally-defined illness, *Asram.***

| **Code (Community No./CG/FGD/Discussion No.): ………/CG/FGD/……**  Day and Date:  Length of the interview:  Place of the interview:  (For each respondent ask the following)  Age:  Occupation:  Level of Education:  Marital status:  Number of children under 5:  Religion:  Ethnic group: |
| --- |

| **Introduction:**  Good morning! My name is ……………., a Research Assistant from the School of Public Health-Kwame Nkrumah University of Science and Technology. This is my colleague……. He/she will be taking some notes during our discussion.  I am here to have a little discussion with you on ‘*Asram*’ disease. The information from this discussion will help in planning for future interventions to reduce child under-5 morbidity and mortality.  This conversation is very confidential and will not be linked to you in any way as I will not ask for your names, but use a code to identify you. I would like to record the discussion using a tape recorder so that I do not miss out on any important information.  This recorded information is for research purposes only and will not be given out to anyone who is not a member of the research team. After we have written out the conversation, it will be erased completely from the memory of the tape recorder.  Does everyone agree to the recording of this discussion? (confirm that all participants consent).  Please take turns while speaking, and do not interrupt anyone. Whatever each one of you shares is of great interest to us, so please be respectful of each other’s opinions. This discussion will last for about 30-45minutes.  Before we start, does anyone have any questions? |
| --- |

|  | **KNOWLEDGE OF CHILDHOOD ILLNESSES AND ASRAM** |
| --- | --- |
| 1. | What are the childhood illnesses that affect children under-5 in this community? |
| 2 | Which of these illnesses cause deaths among children? (If *Asram* is mentioned, don’t ask the next question, ask Q4) |
| 3 | Have your heard of *Asram*? (Probe: from whom? When did you first hear of *Asram*?) |
| 4 | What is *Asram*? (Probe for more definitions and meaning to the disease) |
| 5. | What do you personally know about *Asram*? (Probe: Have you has a child with *Asram*? If yes, probe further how and when? |
| 6. | What is your belief regarding this illness? (Probe: Is it still existent? Does it continue to affect the lives of children? |
|  | **TYPES OF ASRAM** |
| 7. | What are the types of *Asram* you know or have heard of? |
| 8. | May I know the symptoms of the *Asram* types you have mentioned? |
| 9. | What causes these types of *Asram*? (probe for multiple symptoms) |
| 10. | Which types are severe? (Probe: what makes some types more severe than others) |
|  | **MANAGEMENT AND TREATMENT OF ASRAM** |
| 11. | Who treats this disease? (Probe for reasons for choices of treatment) (*Asram* healers versus hospital) |
| 12. | How is *Asram* in a child under 5 treated? (Probe for different treatment and management options) |
|  | **EFFECTS OF ASRAM AMONG CHILDREN ON COMMUNITIES AND COUNTRY** |
| 13. | What are the effects of *Asram* on the child, the entire family and Ghana at large? |
| 14. | How would you like Ghana Health Service to handle this disease? (Probe for specific recommendations) |
| 15. | What advice would you give to mothers/caregivers, pregnant women, etc on this disease? |
| 16. | Do you have anything more to add to what we have said concerning *Asram*? |
