# Supplementary material for: ‘This disease is not meant for the hospital, it is Asram’: Implications of a traditionally-defined illness on healthcare seeking for children under-5 in rural Ashanti, Ghana
Source: PLOS Glob Public Health. 2022 Sep 8;2(9):e0000978. doi: 10.1371/journal.pgph.0000978 (PMC10021330; doi:10.1371/journal.pgph.0000978)
Supplement: S4 Appendix — (DOCX) [file pgph.0000978.s004.docx]

**Key Informant Interview(KII) guide for *Asram* healers on the traditionally-defined illness, *Asram***

| **Code:(Community No./AH/KII/Interview no.) ……../AH/KII.........**  Day and Date:  Length of the interview:  Place of the interview:  (Ask *Asram* healer the following:)  Age:  Occupation:  Level of Education:  Marital status:  Number of children under 5:  Religion:  Ethnic group: |
| --- |

| **Introduction:**  Good morning! My name is ……………., a Research Assistant from the School of Public Health-Kwame Nkrumah University of Science and Technology. This is my colleague……. He/she will be taking some notes during our interview.  I am here to have a little discussion with you on ‘*Asram*’ disease. The information from this discussion will help in planning for future interventions to reduce child under-5 morbidity and mortality in Ghana.  This conversation is very confidential and will not be linked to you in any way as I will not ask for your name, but use a code to identify you. I would like to record the discussion using a tape recorder so that I do not miss out on any important information.  This recorded information is for research purposes only and will not be given out to anyone who is not a member of the research team. After we have written out the conversation, it will be erased completely from the memory of the tape recorder.  Do you agree to the recording of this conversation? This discussion will last for about 30-45minutes.  Before we start, do you have any questions? |
| --- |

| 1. | What are the childhood illnesses that affect children under-5 in this community? |
| --- | --- |
| 2 | Which of these illnesses cause deaths among children? |
| 3 | When did you first hear of *Asram*? (Probe: from whom? Where?) |
| 4 | What exactly is this disease *Asram*? (Probe for more definitions and meaning to the disease) |
| 5. | How long have you been an *Asram* healer? |
| 6. | How did you become a healer? May I know the story of your journey to becoming a popular *Asram* healer? (probe: who, how, when?) |
| 7. | What are the types of *Asram* in this area? |
| 8. | May I know the symptom*s* of the *Asram* types you have mentioned? (Probe for more symptoms and characteristics) |
| 9. | What are the causes of these types of *Asram*? (probe for multiple causes) |
| 10. | What are specific types you treat? |
| 11. | Which one(s) are you specialised in? |
| 12. | Which types do you consider to be severe? |
| 13. | How do you treat *Asram*? |
| 14. | Could you describe the treatment and management processes for your clients? |
| 15. | Do you refer clients to the hospital? |
|  | On average, how many children do you treat each month? (Probe for successful treatments) |
| 16. | Have you been unsuccessful with some treatments? (Probe: How many children have died after unsuccessful treatment) |
| 17. | What is your view about caregivers seeking treatment for *Asram* in the hospital first? |
| 18. | What are the effects of *Asram* on the child, the entire family and Ghana at large? |
| 19. | How would you like Ghana Health Service to handle this disease? (Probe for specific recommendations) |
| 20. | What advice would you give to mothers/caregivers, pregnant women, etc on this disease? |
| 21. | Do you have anything more to add to what we have said concerning *Asram*? |
