# Supplementary material for: ‘This disease is not meant for the hospital, it is Asram’: Implications of a traditionally-defined illness on healthcare seeking for children under-5 in rural Ashanti, Ghana
Source: PLOS Glob Public Health. 2022 Sep 8;2(9):e0000978. doi: 10.1371/journal.pgph.0000978 (PMC10021330; doi:10.1371/journal.pgph.0000978)
Supplement: S6 Appendix — (DOCX) [file pgph.0000978.s006.docx]

**Summary of types of *Asram*, description of their symptoms, and treatment approaches**

| **Type of *Asram*** | **Description of symptoms** | **Perceived cause** | **Treatment approaches** |
| --- | --- | --- | --- |
| *Asram* *Boredwo* | Emancipated body  Stunted growth | Eating roasted plantain during pregnancy is believed to result in preterm birth. | *Asram* herbs  *Asram* bath  Chameleon baptism  *Asram* herbs made up of either leaves, barks, roots, or branches of some trees  *Asram* bath  Wall gecko bracelets or necklaces  The bark of a tree boiled and served as medicine for child |
| *Asram ntoos* | Multiple blisters on the child’s skin | Contact with a spider web during pregnancy  Eating the meat of a squirrel  Eating hot meals during pregnancy |  |
| *Asram borfre* | Bigger head compared to the body size  Excessively soft skin  Incessant crying | Eating pawpaw during pregnancy |  |
| *Asram mpompo* | Rashes and blisters on baby’s skin  Fever | Passed on to baby by *Asram* healer |  |
| *Asram mapaemu* | Fissures in the skull of a baby  Sunken fontanel | Spiritual eye (envious neighbour) |  |
| *Asram ayamtuo* | Bloated stomach  Greenish watery stool for a period  Weight loss | Passed on from mother to baby during pregnancy  Punishment for eating okra during pregnancy |  |
| *Asram esuro* | A sudden spike in temperature  Fever for a number of days  Convulsion | Spiritual eye |  |
| *Asram nofo-denden* | Engorged and painful breasts of a lactating mother.  Passes Asram to baby through breastmilk from the engorged breasts. This makes the baby unable to suckle. | Punishment for a pregnant woman who cheats on her husband |  |
| *Asram pepe* | Baby’s skin turns blue  Struggling to breathe or breathing excessively fast | Prolonged labour  Baby unable to cry at birth |  |
